# Does time matter? Intraspecific diversity of ribosomal RNA genes in lineages of the allopolyploid model grass *Brachypodium hybridum* with different evolutionary ages

**DOI:** 10.1186/s12870-024-05658-5

**Published:** 2024-10-18

**Authors:** Dana Trunova, Natalia Borowska-Zuchowska, Serhii Mykhailyk, Kai Xia, Yuanbin Zhu, Ruben Sancho, Magdalena Rojek-Jelonek, Sònia Garcia, Kai Wang, Pilar Catalan, Ales Kovarik, Robert Hasterok, Bozena Kolano

**Affiliations:** 1 Plant Cytogenetics and Molecular Biology Group, Institute of Biology, Biotechnology and Environmental Protection, Faculty of Natural Sciences, University of Silesia in Katowice, Katowice, 40-032 Poland; 2 School of Life Sciences, Nantong University, Nantong, Jiangsu 226019 China; 3 Department of Agricultural and Environmental Sciences, High Polytechnic School of Huesca, University of Zaragoza, Huesca, 22071 Spain; 4 Institut Botànic de Barcelona IBB (CSIC-CMCNB), Barcelona, Catalonia 08038 Spain; 5 Department of Molecular Epigenetics, Institute of Biophysics, Czech Academy of Sciences, Brno, CZ- 61200 Czech Republic

**Keywords:** rDNA loci, *Brachypodium hybridum*, 5S rDNA NTS, nrITS, 35S rDNA IGS, FISH

## Abstract

**Background:**

Polyploidisation often results in genome rearrangements that may involve changes in both the single-copy sequences and the repetitive genome fraction. In this study, we performed a comprehensive comparative analysis of repetitive DNA, with a particular focus on ribosomal DNA (rDNA), in *Brachypodium hybridum* (2*n* = 4*x* = 30, subgenome composition DDSS), an allotetraploid resulting from a natural cross between two diploid species that resemble the modern *B. distachyon* (2*n* = 10; DD) and *B. stacei* (2*n* = 20; SS). Taking advantage of the recurrent origin of *B. hybridum*, we investigated two genotypes, Bhyb26 and ABR113, differing markedly in their evolutionary age (1.4 and 0.14 Mya, respectively) and which resulted from opposite cross directions. To identify the origin of rDNA loci we employed cytogenetic and molecular methods (FISH, gCAPS and Southern hybridisation), phylogenetic and genomic approaches.

**Results:**

Unlike the general maintenance of doubled gene dosage in *B. hybridum*, the rRNA genes showed a remarkable tendency towards diploidisation at both locus and unit levels. While the partial elimination of 35S rDNA units occurred in the younger ABR113 lineage, unidirectional elimination of the entire locus was observed in the older Bhyb26 lineage. Additionally, a novel 5S rDNA family was amplified in Bhyb26 replacing the parental units. The 35S and 5S rDNA units were preferentially eliminated from the S- and D-subgenome, respectively. Thus, in the more ancient *B. hybridum* lineage, Bhyb26, 5S and 35S rRNA genes are likely expressed from different subgenomes, highlighting the complexity of polyploid regulatory networks.

**Conclusion:**

Comparative analyses between two *B. hybridum* lineages of distinct evolutionary ages revealed that although the recent lineage ABR113 exhibited an additive pattern of rDNA loci distribution, the ancient lineage Bhyb26 demonstrated a pronounced tendency toward diploidisation manifested by the reduction in the number of both 35S and 5S loci. In conclusion, the age of the allopolyploid appears to be a decisive factor in rDNA turnover in *B. hybridum*.

**Supplementary Information:**

The online version contains supplementary material available at 10.1186/s12870-024-05658-5.

## Introduction

Polyploidy is among the major forces driving plant evolution and is often linked to the origin of key innovations found in many plant lineages [1, 2]. An ancient whole genome duplication (WGD) preceded angiosperm diversification, and further polyploidy events can be traced back to the origin and diversification of the major flowering plant lineages [3, 4]. Among polyploids, two groups are distinguished: (i) autopolyploids, which have more than two of the same genomes in their nuclei (e.g., autotetraploid *Arabidopsis arenosa*) [5], and (ii) allopolyploids, which have a hybrid origin and contain two or more duplicated different genomes, e.g., allohexaploid *Triticum aestivum* and allotetraploid *Chenopodium quinoa* [6, 7]. Both autopolyploid and allopolyploid taxa may arise several times from the same or highly similar parents [8–12]. Multiple origins of the same species can result in karyotypic, genomic, transcriptomic and phenotypic variation across lineages, as demonstrated in recently originated allotetraploid species of *Tragopogon* and *Achillea* (Asteraceae) [11, 13]. Several model plant polyploid systems have been used to study different aspects of post-polyploidisation diploidisation, which leads to rapid and extensive genomic changes, such as genome downsizing, structural chromosome rearrangements, amplifications and/or reactivation of repetitive elements, modification of the gene expression patterns, and concerted evolution of multigene families, e.g., ribosomal RNA (rRNA) genes [14–20].

A gradual evolution of a polyploid genome has been shown for the model grass system of the natural allotetraploid *Brachypodium hybridum* (2*n* = 4*x* = 30, subgenome composition DDSS) and two modern diploid species that likely resemble its ancestors: *B. distachyon* (2*n* = 2*x* = 10, DD) and *B. stacei* (2*n* = 2*x* = 20, SS) [21–24]. The phylogenetic studies based on the chloroplast DNA of *B. hybridum* revealed that at least two independent hybridisation events have occurred between *B. distachyon* and *B. stacei* [22]. Crosses between progenitor species occurred in both directions; therefore, two lineages of *B. hybridum* were distinguished: (i) plastotype with the chloroplast DNA (cpDNA) derived from *B. stacei* (S-plastome), and (ii) plastotype with the cpDNA derived from *B. distachyon* (D-plastome) [22, 25–27]. The *B. hybridum* lineages with ancestral D-plastome and S-plastome were formed at approximately 1.4 and 0.14 Mya, here represented by accessions Bhyb26 and ABR113, respectively. Multiple lines of evidence suggest that *B. hybridum* plants with D- and S-plastomes are reproductively isolated [22]. A high level of collinearity between the genomes of the evolutionary younger *B. hybridum* (S-plastome) and the diploid *B. distachyon* and *B. stacei* was shown [22, 26–28], while the older lineage (D-plastotype) was characterised by some gene loss and few genome rearrangements when compared with the younger lineage (*B. hybridum* ABR113) and diploid species [22, 27]. No trace of homoeologous recombination and significant mobile element activation was found for both hybrid lineages [26]. However, there was evidence implying post-polyploidisation transposon activity in them (*B. hybridum*) [26, 27]. Many analyses reported that allopolyploids often preferentially retain genes from one dominant subgenome [29]. When considering the single-copy genes only, neither of the *B. hybridum* genotypes showed genome dominance at the transcriptional level; however, a biased gene loss appeared stronger in the older lineage [22, 27], although it may have resulted from the lack of contemporary progenitor genomes for an accurate comparative analysis [26].

In eukaryotes, housekeeping genes include the tandemly organised rRNA genes (35S rRNA genes and 5S rRNA genes) [30]. Each unit of 35S rDNA contains conserved genic regions encoding for 18S–5.8S-25S and intergenic spacer (IGS) and two internal transcribed spacers (ITS1 and ITS2) [31]. The 5S rDNA units are usually distributed in separate locus/loci in the genome and consist of the conserved genic region and variable non-transcribed spacer (5S rDNA NTS) [31, 32]; however, the linked rDNA configuration (in which the 5S rDNA is inserted into the 35S rDNA IGS) was also described [33, 34]. The evolutionary pathways of rRNA genes may not be the same as the single-copy ones, as was shown indeed in *B. hybridum* ABR113, where a significant reduction of the S-subgenome rDNA units was observed [35] while there was a balanced composition and expression of single-copy genes [22, 26]. Previous studies also revealed that the *B. stacei*-like 35S rDNA loci were transcriptionally silenced in all studied tissues of this genotype [36–38], showing a strong nucleolar dominance (ND) towards the D-subgenome rDNA. Until now, stable ND has been confirmed in a wide range of *B. hybridum* genotypes originating from different climatic zones [35]. Recent studies on another *B. hybridum* genotype (3-7-2; new name: Bhyb3_7_2) revealed tissue-specificity of ND in this genotype [39]. The evolutionary patterns of 35S rRNA genes have not been studied yet in *B. hybridum* Bhyb26. Also, little is known about the 5S rDNA evolution in this species. In most genotypes, both ancestral 5S rDNA loci are present [35, 40, 41]. However, previous work detected a significant reduction of S-genome 5S rDNA copies in one of the genotypes using a bioinformatics approach [40].

The current study draws a complex picture of rRNA gene evolutionary trajectories in two *B. hybridum* lineages of distinct evolutionary ages and cross directions. We aimed to address the following questions: (i) are the evolutionary trajectories of 35S and 5S rDNA homoeologues similar?; (ii) is there a relationship between the direction of the cross and rDNA homoeologues loss in *B. hybridum*?; (iii) do the rRNA genes in the studied *B. hybridum* lineages of different evolutionary ages follow the same evolutionary pathways?

## Materials and methods

### Plant material

Seven genotypes of the three annual *Brachypodium* species were analysed (Table 1). Plants used for DNA isolation were grown from seeds in pots with soil mixed with vermiculite (3∶1 v/v) at 20 °C and 16/8 h photoperiod in the greenhouse of the University of Silesia in Katowice, Poland. For cytogenetic analyses, the primary roots of the seedlings were used. Seeds were germinated on a filter paper moistened with water for 3–5 days at 20–22 °C in the dark. Whole seedlings with approximately two cm-long roots were placed in ice-cold water overnight and fixed in 3:1 (v/v) methanol: glacial acetic acid.

**Table 1:** General characteristics of the *Brachypodium* species used in this study

Species	Genotype	2*n*	*x*	Genome /subgenome designation	Origin	Source
*Brachypodium distachyon* (L.) P. Beauv.	Bd21-3	10	5	DD	Iraq	A
	Bd21	10	5	DD	Iraq	A
	ABR5	10	5	DD	Spain, Huesca	B
*Brachypodium stacei* Catalán, Joch. Müll., Mur & Langdon	ABR114	20	10	SS	Spain, Formentera	B
	Bsta5	20	10	SS	Spain, Alicante	C
*Brachypodium hybridum* Catalán, Joch. Müll., Hasterok & Jenkins	ABR113	30	10 + 5	DDSS	Portugal, Lisbon	B
	Bhyb26	30	10 + 5	DDSS	Spain, Jaen, La Cimbarra	C

A, US Department of Agriculture, National Plant Germplasm System, Beltsville, MD, USA; B, Institute of Biological, Environmental and Rural Sciences, Aberystwyth University, Aberystwyth, UK; C, High Polytechnic School of Huesca, University of Zaragoza, Huesca, Spain

### NGS data and in silico identification of rDNA repeats

The raw Illumina sequencing data for *B. distachyon* Bd21-3 (SRR4236817), *B. stacei* ABR114 (SRR1800504), *B. hybridum* ABR113 (SRR3945061) and *B. hybridum* Bhyb26 (SRR4184872), available in the European Nucleotide Archive (ENA), were utilised to identify rDNA repeats. Depending on the sequencing library, the paired-end read length ranged from 150 to 250 bp. The Illumina reads were trimmed to a length of 150 bp, and the high quality of the trimmed reads was ensured by the QC tools of RepeatExplorer2 [42, 43]. All libraries were sampled at random to obtain 1× coverage of the genome. A graph-based clustering of paired-end reads was performed to identify, characterise and quantify repetitive sequences with the web-based RepeatExplorer2 pipeline implemented in the Galaxy server [https://galaxy-elixir.cerit-sc.cz/; 42]. The reads were analysed using default parameters (90% similarity, minimum overlap = 55; cluster size threshold = 0.01%) in each *Brachypodium* genotype separately [42, 43].

### DNA isolation, polymerase chain reaction and cloning

Total genomic DNA (gDNA) was isolated from young and healthy leaves of 1-month-old plants using a cetyltrimethylammonium bromide-based (CTAB) method [44]. To analyse the ancestral contribution of rDNA variants in the allotetraploid, the nrITS, IGS of 35S rDNA and 5S rDNA NTS were cloned and sequenced. nrITS (ITS1-5.8S rDNA-ITS2) was amplified using a primer pair anchored in 18S rDNA and 25S rDNA (18Sdir and 25Scom; Table S1) [45] as earlier described [46]. The PCR product was treated with *E. coli* Exonuclease I and FastAP Thermosensitive Alkaline Phosphatase (Thermo Fisher Scientific, Waltham, USA) according to the manufacturer’s instruction, and the cycle sequencing was performed using a 3730xl DNA Analyzer (Applied Biosystems; USA) in a commercial facility (Macrogen, Amsterdam, Netherlands). The 35S rDNA intergenic spacer of *B. hybridum* genotype Bhyb26 was amplified from the gDNA using a primer pair designed to match the conserved regions of the 18S and 25S rRNA genes (Table S1) [47] as described [36]. Amplification products were separated by electrophoresis on a 1% agarose gel stained with GelRed (Sigma-Aldrich, Steinheim, Germany) and visualised with a UV transilluminator. PCR products were gel-purified using the QIAquick Gel Extraction Kit (Qiagen, Germany). The amplicons were cloned using the pGEM-T Easy vector system (Promega, Madison, USA) following the manufacturer’s instructions. Four clones were sequenced using the Sanger method and the primer-walking strategy in a commercial facility (Genomed, Warsaw, Poland). The 5S rDNA non-transcribed spacer (5S rDNA NTS) was amplified using a primer pair anchored in the 5S rRNA genic region (Table S1) [48] as earlier described [7]. PCR products were gel-purified as described above, and ligated into pGEM-T Easy vectors (Promega, Madison, USA) following the manufacturer’s instructions. Depending on the ploidy level, five to ten randomly chosen recombinant colonies were selected for further analyses, and the universal primer M13 was used for sequencing. Plasmid DNA was isolated using a QIAGEN Plasmid Mini Kit according to the manufacturer’s instructions. Sanger sequencing was performed in a commercial facility (Macrogen, Amsterdam, Netherlands). All the newly generated sequences were deposited in GenBank: 5S rDNA NTS (PP339782- PP339812, PP339815, PP339816, PP339825), 35S rDNA IGS (PP339781) and nrITS (PP317522; Table S2).

### Bioinformatic and phylogenetic analyses

Comparative analyses were performed to assess the contribution of ancestral variants in both analysed allotetraploid lineages and to reveal the direction of rDNA sequence evolution in this tetraploid. Newly obtained sequences were assembled using CLC Genomics Workbench (Qiagen, Hilden, Germany). Multiple sequence alignments were performed in Geneious Prime 2022.0.1 (https://www.geneious.com) and manually adjusted. Comparative analysis of 35S rDNA IGS among analysed genotypes was done using newly obtained sequences and those published earlier (GenBank accession numbers: KX263276, KX263278, KX26327). Multiple sequence alignments for the 35S rDNA IGS were manually adjusted in Geneious Prime 2022.0.1.

The sequence of the cloned IGS from Bhyb26 was used as the reference sequence in the *B. hybridum* Bhyb26 raw Illumina reads mapping using the ‘Map Read Reference’ tool in the CLC Genomics Workbench. All reads containing ambiguous nucleotides, i.e. that were shorter than 150 bp and/or failed to pass a quality score limit of *P* = 0.05, were removed using the ‘TRIM’ command in CLC Genomics Workbench. The mapping parameters were as follows: mismatch cost, 2; insertion cost, 3; deletion cost, 3; with the length fraction set at 0.5 and the similarity fraction set at 0.8. The consensus IGS sequences were then extracted from the mapped reads. Additionally, the 35S rDNA IGSs were compared using the DOTTER and Tandem Repeats Finder [49, 50].

Multiple sequence alignments for the 5S rDNA NTS dataset (in total 44 sequences; Table S2) were performed using webPRANK [51] and manually adjusted. Phylogenetic relationships for 5S rDNA NTS regions were inferred using maximum likelihood (ML), as implemented in IQ-TREE version 2.2.2 with default parameters [52, 53]. The best model of sequence evolution for the ML analysis, K2P, was determined using the Bayesian information criterion as implemented in IQ-TREE. The significance of the inferred relationships was assessed via bootstrapping with 1000 replicates. The resulting phylogenetic tree was visualised using FigTree v.1.3.1. The copy numbers of the 35S rDNA and 5S rDNA units were calculated from the NGS read count using the following scheme: (i) the genome proportion (GP) was calculated from the number of mapped reads to either the 18S or 5S rDNA consensus sequence divided by the total number of reads in percentages; (ii) the genome spaces (GS) of particular rDNAs were determined using the following formula: GP × size of the genome in Mb (618 Mb and 650 Mb for ABR113 and Bhyb26, respectively); (iii) the copy numbers of rDNAs were calculated as follows: GS value divided by the size of a either 18S rDNA in Mb (0.00181 Mb) or 5S rDNA coding region (0.000119 Mb).

### Restriction analyses (gCAPS) and Southern blot hybridisation

In order to determine the ancestral contribution of rDNA variants in studied lineages of *B. hybridum*, the following approaches have been performed: (i) the genomic cleaved amplified polymorphic sequence (gCAPS) of ITS1 region, (ii) Southern hybridisation with 25S rDNA and (iii) 5S rDNA as a probe. The genomic cleaved amplified sequence polymorphism analyses (gCAPS) were performed as described in [35]. Shortly, the ITS1 region of the 35S rRNA gene was amplified using a primer pair 18S-for and 5.8S-rev [Supplementary Table S1; 54]. The PCR products were then digested with a *Mlu*I restriction enzyme (New England Biolabs, Ipswich, USA) and size separated on 1% (w/v) agarose gel.

Southern blot hybridisation with 5S rDNA was performed using a non-radioactive method. The genomic DNA (2 µg) from *Brachypodium* spp. were subjected to digestion with *Mse*I enzyme (New England Biolabs, Ipswich, USA) at 37 °C for 4 h. The digested gDNAs were separated on 1.2% (w/v) agarose gel and blotted onto positive-charged nylon membranes (Roche, Mannheim, Germany) using VacuBlot System (Biometra Analytik Jena, Jena, Germany). DNA was UV cross-linked to the membrane using a CK-1000 Ultraviolet Crosslinker (Ultra-Violet Products, Cambridge, UK). The 5S rDNA monomer of *Arabidopsis thaliana* [54] labelled with digoxigenin-11-dUTP, alkali-labile, using nick translation according to manufacturer’s instructions (Roche, Basel, Switzerland) was used as a probe. Hybridisation was performed using a DIG High Prime DNA Labeling and Detection Starter Kit II (Roche, Mannheim, Germany) according to the manufacturer’s instructions using an HB-1000 Hybridizer (UltraViolet Products, Cambridge, UK). Hybridisation signals were documented and quantified using ChemiDocXRS (BioRad, USA).

The Southern blot hybridisation of 35S rDNA was carried out following the methodology earlier described [55]. The genomic DNA (1 µg) was treated with *Bgl*II restriction enzyme (New England Biolabs, Ipswich, USA) at 37°C for 2.5 h. Afterwards, the samples were loaded onto a 1% agarose gel, separated through electrophoresis, and transferred to a nylon membrane (Amersham Hybond, GE Healthcare, USA). The membrane was then hybridised using a 25S rRNA gene labelled with [α-^32^P]dCTP as a probe, a 220 bp PCR product derived from the 3’ end of the 25S rDNA from *Nicotiana tabacum*. The hybridisation was visualised using a phosphoimager (Typhoon 9500, GE Healthcare, USA), and the intensity of the 35S rDNA signal was measured using ImageQuant software (GE Healthcare, USA). Two biological replicates were performed for each probe.

### Chromosome preparation and fluorescence in situ hybridisation (FISH)

The FISH method with 25S and 5S rDNA as probes was used to analyse the rRNA gene loci number and chromosomal localisation in the studied *Brachypodium* genotypes. The chromosome preparations were made following the published protocol [41]. A 2.3-kb fragment of the 25S rDNA coding region of *A. thaliana* [56] labelled with digoxigenin-11- dUTP (Roche, Basel, Switzerland) was used to detect 35S rDNA loci. The 5S rDNA monomer isolated from *T. aestivum* [54] and labelled with tetramethyl-rhodamine-5-dUTP (Roche, Basel, Switzerland) was used to detect the 5S rDNA loci. Both probes were labelled using nick translation according to the manufacturer’s instructions (Roche, Basel, Switzerland). FISH was performed as previously described [41]. The hybridisation mixture consisting of 50% deionised formamide, 10% dextran sulphate, 2× SSC, 0.5% SDS (sodium dodecyl sulphate) and 100 ng of each labelled DNA probe was denatured for 10 min at 80 °C and immediately cooled down on ice. The denaturation of the slides and the hybridisation mixture were performed on an Omnislide Thermal cycler (ThermoHybaid, Franklin, MA, USA) at 75 °C for 4 min. Hybridisation was conducted for 20 h at 37 °C in a humid chamber. Post-hybridisation washes (10% deionised formamide in 0.1× SSC at 42 °C; stringency 76%) were followed by the immunodetection of digoxigenated probes using FITC-conjugated anti-digoxigenin antibodies (Roche, Basel, Switzerland). The slides were mounted in Vectashield (Vector Laboratories, Newark, CA, USA) containing 2.5 ng/µl of DAPI (4′,6-diamidino-2-phenylindole dihydrochloride). All images were acquired using a Zeiss AxioImager.Z.2 fluorescent microscope equipped with an AxioCamHMr camera (Zeiss, Oberkochen, Germany). The images were processed uniformly using ZEN 2.3 Pro (Zeiss).

## Genome size determination

The genome size of *B. hybridum* Bhyb26 was determined by flow cytometry. Sixteen Bhyb26 individuals, grown from seeds, were analysed and each sample was measured twice. All analyses were performed by the same operator when the plants were 4–5 weeks old; a maximum of five plants were analysed in one working day. The youngest fully developed leaves of the studied *B. hybridum* plants and the internal standard *Lycopersicon esculentum* Mill. cv. Stupicke (2C DNA = 1.96 pg) [57] were chopped together in a Petri dish in 500 µl of a nuclei extraction buffer using a razor blade (Sysmex CyStain PI OxProtect, 05-5027-P01). The nuclei suspension was filtered through a 30 μm mesh (CellTrics, Sysmex, Kobe, Japan) and stained with a staining buffer containing propidium iodide and RNase (Sysmex CyStain PI OxProtect, 05-5027-P01) according to the manufacturer’s instructions. The samples were incubated for 45 min in the dark and then analysed using a flow cytometer (CyFlow Space, Sysmex, Kobe, Japan) equipped with a 532 nm green laser. At least 10,000 nuclei were analysed for each sample. The sizes of the nuclear genomes were calculated as the linear relationship between the ratio of the 2C DNA peaks of a sample and the standard according to the formula:

$$\begin{aligned}&\text{S}\text{a}\text{m}\text{p}\text{l}\text{e}\:2\text{C}\:\text{v}\text{a}\text{l}\text{u}\text{e}\:\left(\text{D}\text{N}\text{A}\:\text{p}\text{g}\:\text{o}\text{r}\:\text{M}\text{b}\right)\\&\quad=\text{R}\text{e}\text{f}\text{e}\text{r}\text{e}\text{n}\text{c}\text{e}\:2\text{C}\:\text{v}\text{a}\text{l}\text{u}\text{e}\times\frac{\text{s}\text{a}\text{m}\text{p}\text{l}\text{e}\:2\text{C}\:\text{m}\text{e}\text{a}\text{n}\:\text{p}\text{e}\text{a}\text{k}\:\text{p}\text{o}\text{s}\text{i}\text{t}\text{i}\text{o}\text{n}}{\text{r}\text{e}\text{f}\text{e}\text{r}\text{e}\text{n}\text{c}\text{e}\:2\text{C}\:\text{p}\text{e}\text{a}\text{k}\:\text{p}\text{o}\text{s}\text{i}\text{t}\text{i}\text{o}\text{n}}\end{aligned}$$

The conversion of pg to Mb was made according to the formula:

DNA content (pg) = genome size (bp)/(0.978 × 10^9^) [58].

## Results

### Number and chromosomal distribution of 35S and 5S rDNA loci

**Fig. 1:**
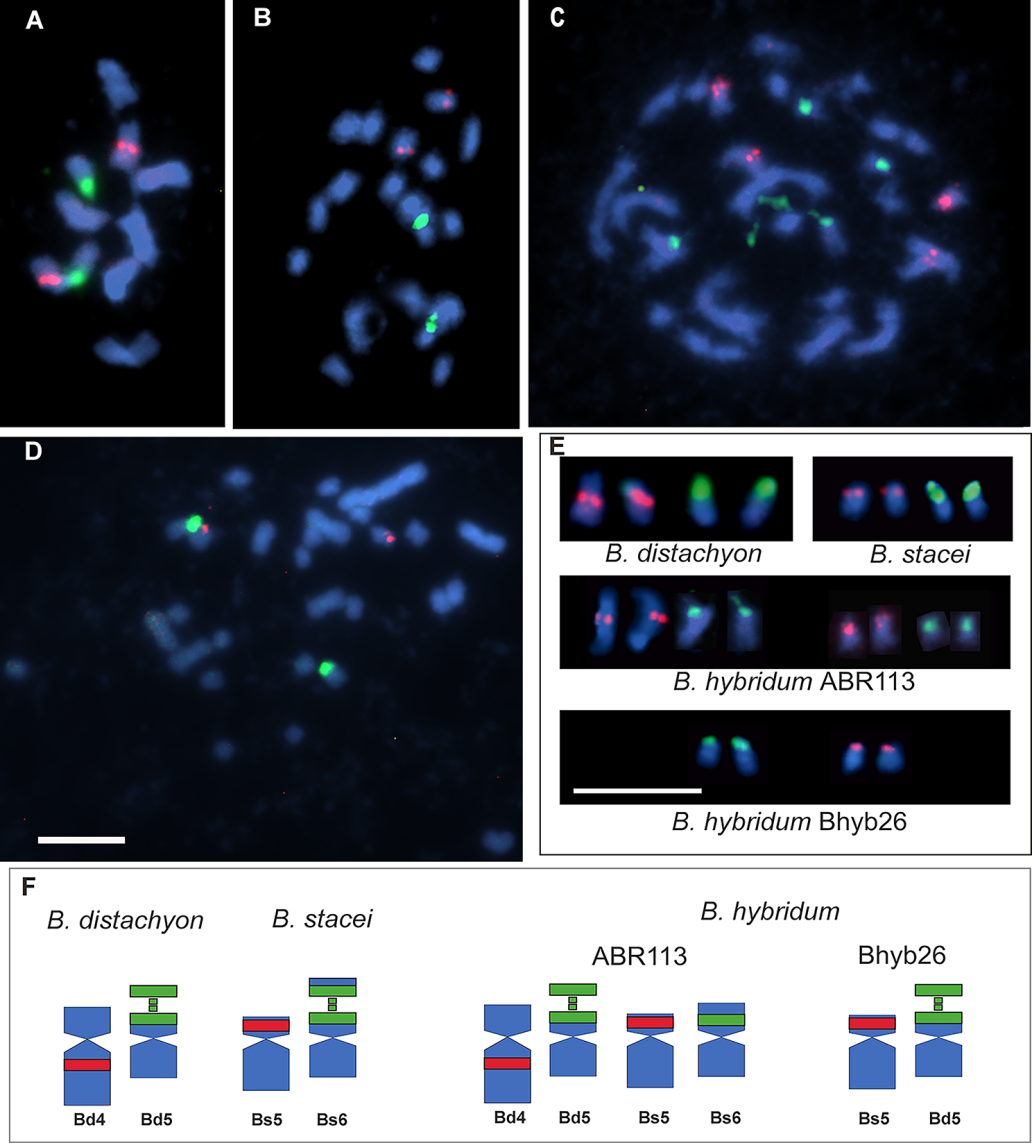
Distribution of the 35S and 5S rDNA loci on the mitotic chromosomes of the studied *Brachypodium* accessions. Fluorescence in situ hybridisation (**A**-**E**) of the 25S rDNA (green fluorescence) and 5S rDNA (red fluorescence) probes to (**A**) *B. distachyon* Bd21 (2*n* = 10; *x* = 5), (**B**) *B. stacei* ABR114 (2*n* = 20; *x* = 10), (**C**) *B. hybridum* ABR113 (2*n* = 30; *x* = 5 + 10) and (**D**) *B. hybridum* Bhyb26 (2*n* = 30; *x* = 5 + 10) chromosomes. (**E**) Chromosomal pairs that bear rDNA loci and (**F**) their idiograms. All bars: 5 μm

The chromosome numbers were verified for all three analysed species. As expected, 2*n* = 10 was observed in meristematic cells of *B. distachyon* and 2*n* = 20, with significantly smaller chromosomes, in *B. stacei*. Both genotypes of *B. hybridum* were characterised with 2*n* = 30, including ten larger chromosomes originating from the *B. distachyon*-like ancestor and 20 smaller ones derived from the *B. stacei*-like ancestor, as previously described [28]. In agreement with previous reports [28, 41], FISH with 5S rDNA and 25S rDNA as probes confirmed that *B. distachyon* has one 5S rDNA locus in the proximal region of the long arm of chromosome Bd4 and one 35S rDNA locus at the distal region of the short arm of chromosome Bd5 (Fig. 1A, E, F). An identical number of loci was confirmed in *B. stacei*, i.e. one 5S rDNA locus in the short arm of chromosome Bs5 and one 35S rDNA locus in the proximal region of the short arm of chromosome Bs6 (Fig. 1B, E and F) [22]. While the evolutionary younger *B. hybridum* genotype ABR113 had an additive number of 35S and 5S rDNA loci (Fig. 1C, E and F) derived from both ancestors, the evolutionary older Bhyb26 lineage, studied here for the first time, revealed only two chromosomal pairs bearing rDNA loci: one derived from the D-subgenome with the 35S rDNA site, and another pair originated from S-subgenome with the 5S rDNA site (Fig. 1D, E and F).

### In silico identification and genome proportion of rDNA repeats

**Fig. 2:**
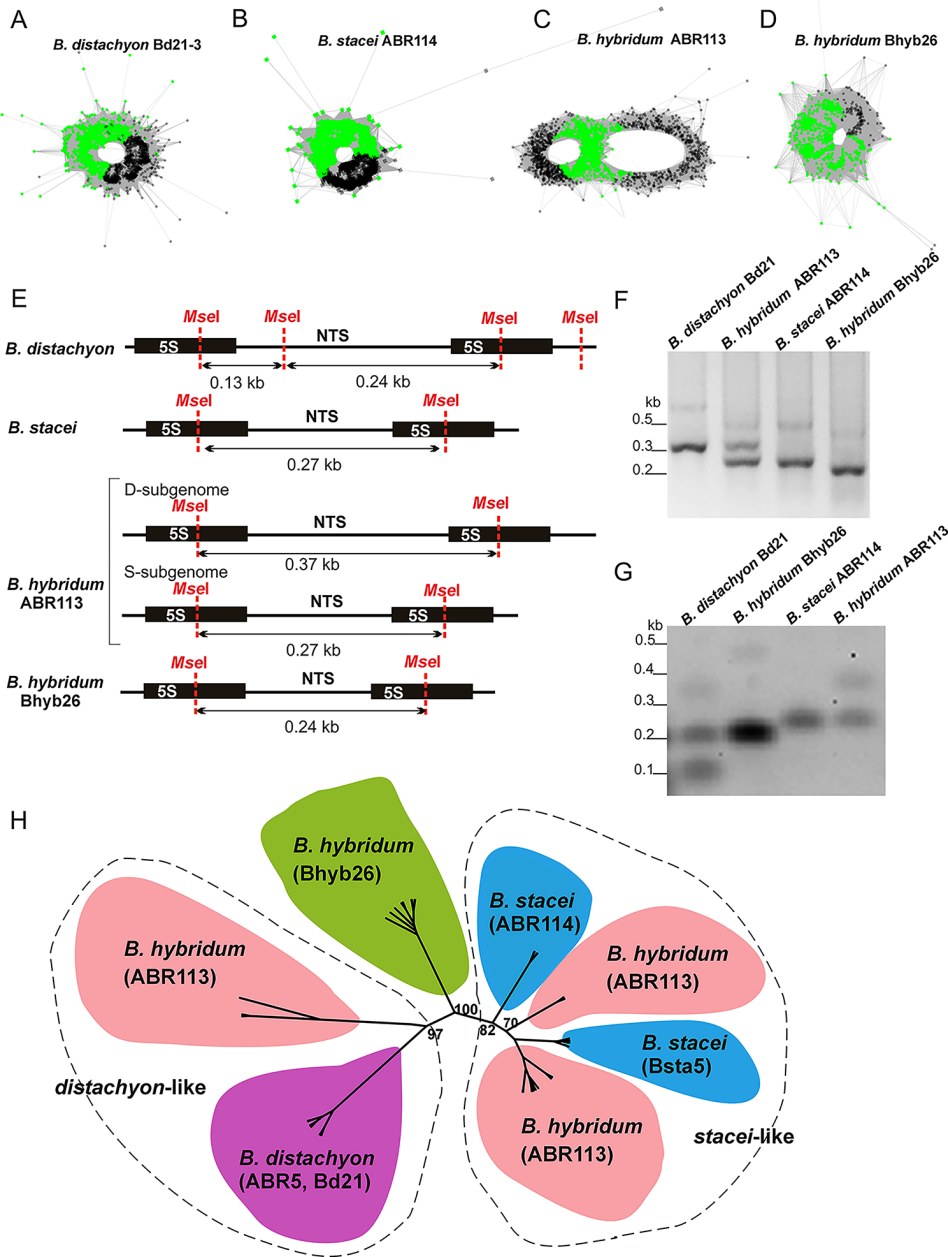
Structure of 5S rDNA in the studied *Brachypodium* accessions. 5S rDNA sequence reads organised in graph structures from the RepeatExplorer2 graphical output of (**A**) *B. distachyon* Bd21-3, (**B**) *B. stacei* ABR114, (**C**) *B. hybridum* ABR113 and (**D**) *B. hybridum* Bhyb26. 5S rDNA coding sequences and intergenic spacers are highlighted as green and grey dots, respectively. Note, simple circle graphs indicate a single 5S rDNA family, and complex two-loop graphs indicate multiple families. (**E**) Length and *Mse*I restriction maps of the 5S rDNA units of the analysed *Brachypodium* accessions. (**F**) Length analysis of 5S rDNA variants. Agarose gel electrophoresis of PCR products consisting of fragments of 5S rDNA coding region (102 bp) and NTSs (the original gel is shown in Supplementary Figure S1). (**G**) Southern blot hybridisation of the *Mse*I-restricted genomic DNA from *B. distachyon*,* B. stacei*,* B. hybridum* ABR113 and Bhyb26 genotypes digested with *Bgl*II and hybridised with the 5S rDNA probe (the original membrane is shown in Supplementary Figure S2). (**H**) The unrooted phylogenetic tree of the studied annual *Brachypodium* species inferred through maximum likelihood analysis of the 5S rDNA NTS alignment. Bootstrap support scores ≥ 70% are shown near branches

The reads complementary to 35S and 5S rDNA sequences were identified using RepeatExplorer2, and the genome proportion of both rDNA sequences was assessed from the raw Illumina reads. One cluster with reads complementary to 5S rDNA was retrieved for each analysed lineage. A simple circular graph was reconstructed for the 5S rDNA cluster in both diploids and *B. hybridum* Bhyb26 (Fig. 2A, B and D). In contrast, in the case of ABR113, a complex graph with two loops interconnected by a junction region (composed of the 5S rDNA coding region) was revealed, indicating the presence of two different NTS variants (Fig. 2C). Based on the raw Illumina reads mapping, the copy number of 5S rDNA was estimated as 1367 units/1C and 2570 units/1C for the younger ABR113 (1C ≈ 618 Mb) and the older Bhyb26 (1C ≈ 650 Mb), respectively (Table 2). The analysed lineages of *B. hybridum* also differed in the genome proportion of 35S rDNA repeats. 35S rDNA accounted for 1356 units/1C and 747 units/1C in ABR113 and Bhyb26, respectively (Table 2).

## 5S rDNA structure

A PCR with primers anchored in coding 5S rDNA sequences was performed on genomic DNA to determine the lengths of 5S rDNA units in *Brachypodium* diploids (parental species) and *B. hybridum* lineages (Fig. 2E and F). The amplicons contained the entire non-transcribed spacer and the 102-bp-long fragment of the coding sequence. The longest PCR product of about 350 bp was obtained for *B. distachyon*, while the *B. stacei* one was much shorter, approximately 250 bp. Two prominent products of about 350 and 250 bp were amplified from the younger *B. hybridum* ABR113, suggesting the presence of two 5S rRNA gene families in this genotype. In contrast, only one prominent band of approximately 220 bp was observed for the older Bhyb26 (Fig. 2F).

**Table 2:** Copy number of the rDNA units in the studied *Brachypodium hybridum* accessions determined from the raw Illumina reads

Species	Genotype	Read archive accession	Mapped reads^a^	Total number of reads	GP [%]^b^	GS^c^	Copies^d^
**18S rDNA**							
*B. hybridum*	ABR113	SRR3945061	7902	2,000,000	0.21	2.44	1356
*B. hybridum*	Bhyb26	SRR4184872	4136	2,000,000	0. 40	1.34	747
**5S rDNA**							
*B. hybridum*	ABR113	SRR3945061	531	2,000,000	0.03	0.16	1367
*B. hybridum*	Bhyb26	SRR4184872	949	2,000,000	0.05	0.31	2570

^a^ The length of the 18S rDNA consensus sequence used as reference was 1.81 kb, and this of 5S rDNA was 119 bp

^b^ The genome proportion (GP) was calculated from the number of mapped reads to the consensus sequence divided by the total number of reads in percentages

^c^ The genome space (GS) was calculated as (genome size in Mb × GP × 100^− 1^)

^d^ The copy number of the rDNA units was calculated as GS/size of a single 18S rDNA unit (0.00181 Mb) or 5S rDNA unit (0.000119 Mb). The following genome sizes were considered: *B. hybridum* ABR113 0.633 pg/1C ≈ 618 Mb (Catalán et al. 2012); *B. hybridum* Bhyb26 0.655 pg/1C ≈ 650 Mb (present study). The conversion of pg to Mb was made according to Dolezel et al. (2003)

The contribution of different length variants of 5S rDNA NTS in genomes of analysed *Brachypodium* lineages was further supported by Southern hybridisation. Bioinformatic analyses of the 5S rDNA sequence revealed the presence of a single restriction site for *Mse*I in *B. stacei* and both allotetraploid genotypes of *B. hybridum* (Fig. 2E). In contrast, there were two sites for this enzyme in the corresponding sequence of *B. distachyon*. After the Southern hybridisation, a single band of approximately 270 bp was observed in *B. stacei*. In *B. distachyon*, the 5S rDNA probe hybridised to two shorter *Mse*I fragments of approximately 240 bp and 130 bp (Fig. 2E and G). Two bands (270 bp and 370 bp) were recovered for *B. hybridum* ABR113 and only a single 240-bp-long band was observed in *B. hybridum* Bhyb26 (Fig. 2E and G). No shorter D-subgenome-specific *Mse*I fragments were visualised in any of the *B. hybridum* accessions.

Maximum likelihood analysis was performed using cloned 5S rDNA NTS from both *B. hybridum* lineages and the two diploid *Brachypodium* annuals. The length of the analysed region ranged from 221 to 356 bp, and the final alignment, used for phylogenetic analyses, was 417 bp long (including gaps) with 70 parsimony informative sites. Among the analysed sequences, three main groups of 5S rDNA NTS were revealed (Fig. 2H). The first well-supported clade (Bootstrap Support 97; BS 97; *distachyon*-like) consisted of sequences isolated from *B. distachyon* and *B. hybridum* ABR113 (the sequences representing the longer variants of 5S rDNA NTS; ~ 353 bp). The second clade (BS 82; *stacei*-like) comprised approximately 250-bp-long sequences isolated from *B. stacei* and *B. hybridum* ABR113. The 5S rDNA sequences isolated from *B. hybridum* Bhyb26 were grouped into the third clade (~ 221 bp; BS 100; Fig. 2H). The *distachon*-like clade could be further divided into two subclades: (i) a subclade composed of sequences isolated from *B. distachyon* and (ii) sequences isolated from *B. hybridum* ABR113. The *stacei*-like clade could be further divided into four subclades. Two of them consisted of sequences isolated from *B. hybridum* ABR113, and two other subclades included sequences isolated from two different accessions of *B. stacei*.

### 35S rDNA structure

**Fig. 3:**
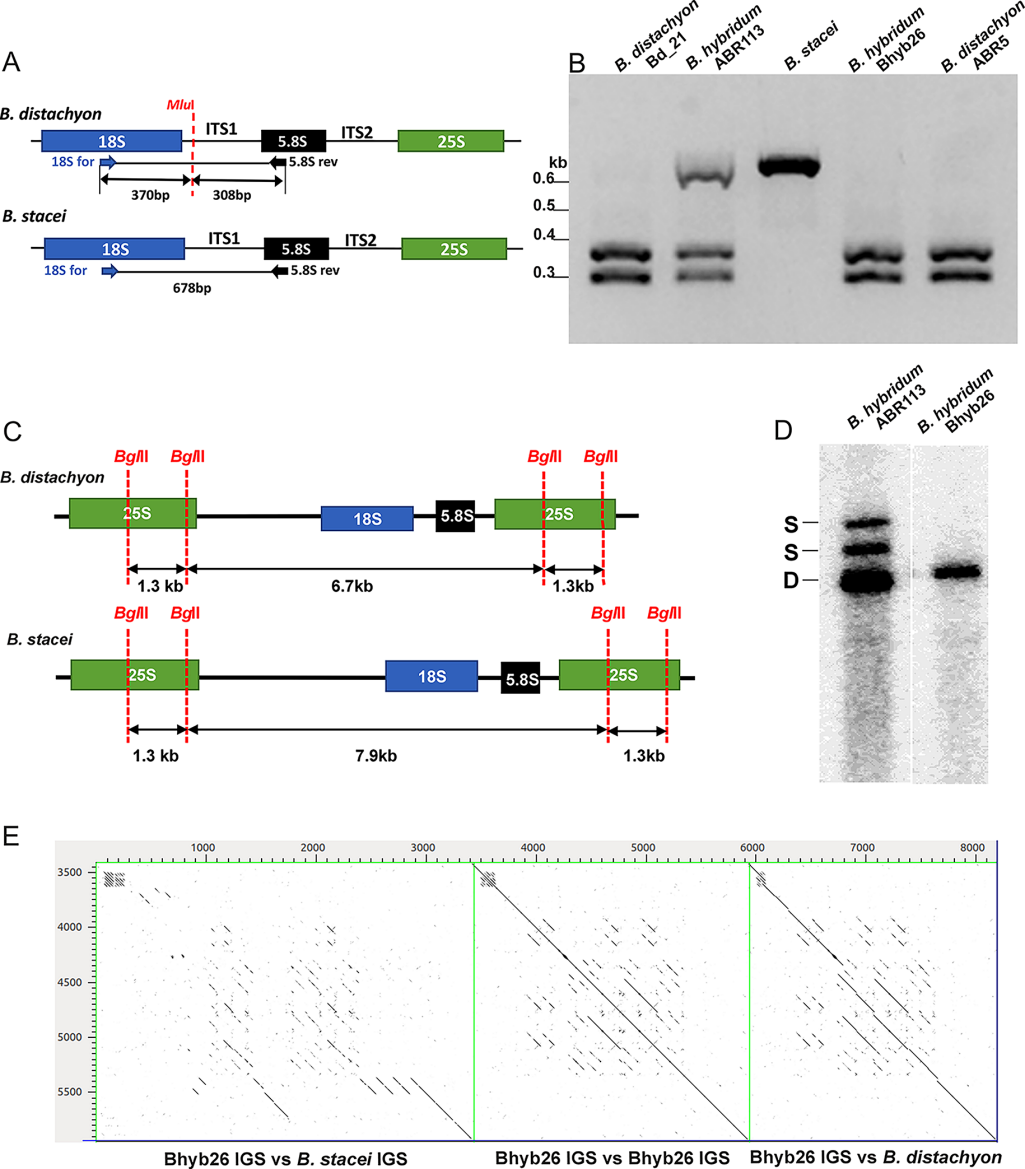
Structure of 35S rDNA in the studied *Brachypodium* accessions. (**A**) *Mlu*I restriction maps of the ITS1 region of the analysed *Brachypodium* accessions. (**B**) The gCAPS analysis of ITS1 sequences amplified from annual *Brachypodium* samples. The fragments were digested with *Mlu*I (the original gel is shown in Supplementary Figure S3). (**C**) *Bgl*II restriction maps of the 35S rDNA units of the analysed *Brachypodium* samples. (**D**) Southern blot hybridisation of genomic DNA from allotetraploid *B. hybridum* lineages digested with *Bgl*II and hybridised with the 25S rDNA probe (the original membrane is shown in Supplementary Figure S4). After hybridisation with 25S rDNA probes, one band (6.7 kb) was shown for the D-subgenome while the S-subgenome was characterised by two bands (9.2 kb and 7.9 kb); a longer one corresponding to the complete repeat (the SNP analysis revealed that one *Bgl*II restriction site could be mutated in some units; [35]) and a shorter one corresponding to the repeat cut two times with *Bgl*II. (**E**) Comparison of the 35S rRNA gene intergenic spacer sequences between *B. hybridum* Bhyb26 and diploid *B. stacei* ABR114 or *B. distachyon* Bd21 on dot matrix plots

The ITS1 and IGS structures of the 35S rDNA homoeologues were analysed in *B. hybridum* younger ABR113 and older Bhyb26 lineages using bioinformatic and molecular biology methods. The polymorphism in the ITS1 of parental diploids allowed to distinguish between the progenitor ITS1 variants in allopolyploids by the gCAPS approach. A single *Mlu*I restriction site was found within the ITS1 of the D-subgenome, while there was no *Mlu*I site in the S-subgenome ITS1 (Fig. 3A). Region containing part of the 3’ end of 18S rDNA together with the whole ITS1 was amplified and subjected to digestion with *Mlu*I. Thus, only the *B. distachyon*-like ITS1 was cut, resulting in two fragments, of approximately 300 and 370 bp, while the *B. stacei* one remained uncut (~ 680 bp). In the younger ABR113, an additive pattern of three expected bands was observed. Interestingly, the older Bhyb26 genotype, showed only the *B. distachyon-*like ITS1 profile (Fig. 3B).

Taking advantage of the length polymorphisms in the progenitor *B. distachyon* and *B. stacei* IGS, it was possible to distinguish between the homoeologous rDNA variants in *B. hybridum* [36]. Bioinformatic analysis showed two restriction sites for *Bgl*II within the 25S rDNA coding region of the 35S rDNA consensus sequences of D- and S-subgenomes [Figs. 3C and 35]. In the case of the younger ABR113 genotype, three *Bgl*II fragments were detected: (i) a fast migrating fragment of 6.7 kb corresponding to D-subgenome rDNA, (ii) a fragment of 7.9 kb corresponding to S-subgenome rDNA, and (iii) a 9.2 kb long fragment representing the complete 35S rDNA unit from the S-subgenome in which a single *Bgl*II site was mutated thus not cut. Only one *Bgl*II fragment of *B. distachyon* origin was observed in the evolutionary older Bhyb26 genotype, showing the elimination of S-subgenome rDNA homoeologues (Fig. 3D).

The IGS from the older *B. hybridum* Bhyb26 genotype was cloned, sequenced and compared with the corresponding regions from *B. stacei* and *B. distachyon*. The length of the IGS in Bhyb26 was 2519 bp. Sequence comparison has shown 86% identity between the IGS regions from *B. distachyon* and Bhyb26; however, two 51 bp- and 29 bp-long subregions of IGS showed similarities to corresponding fragments in *B. stacei*, and three subregions (120 bp-, 12 bp- and 35 bp-long fragments) were Bhyb26-specific (Figure S5). Sequence analysis with both dot matrix plot and Tandem Repeats Finder revealed that Bhyb26 IGS contained internal repetitive motifs that resemble the *B. distachyon* 35S rDNA IGS, both in the sequence and organisation context (Fig. 3E and Figure S6). It is worth noting that the IGS contained several TATA box elements corresponding to genic and spacer promoters. The putative transcription initiation site immediately downstream of the TATA element showed high identity with its counterparts from diploid *Brachypodium* species. The sequence conservation of the Bhyb26 IGS was in silico verified by Bhyb26 raw Illumina reads mapping to the Bhyb26 cloned IGS, followed by the extraction of the consensus IGS sequence and re-mapping of reads and SNP analysis. Interestingly, only three SNPs were found in the ETS (SNP frequency set as ≥ 10%, Figure S6).

## Discussion

**Fig. 4:**
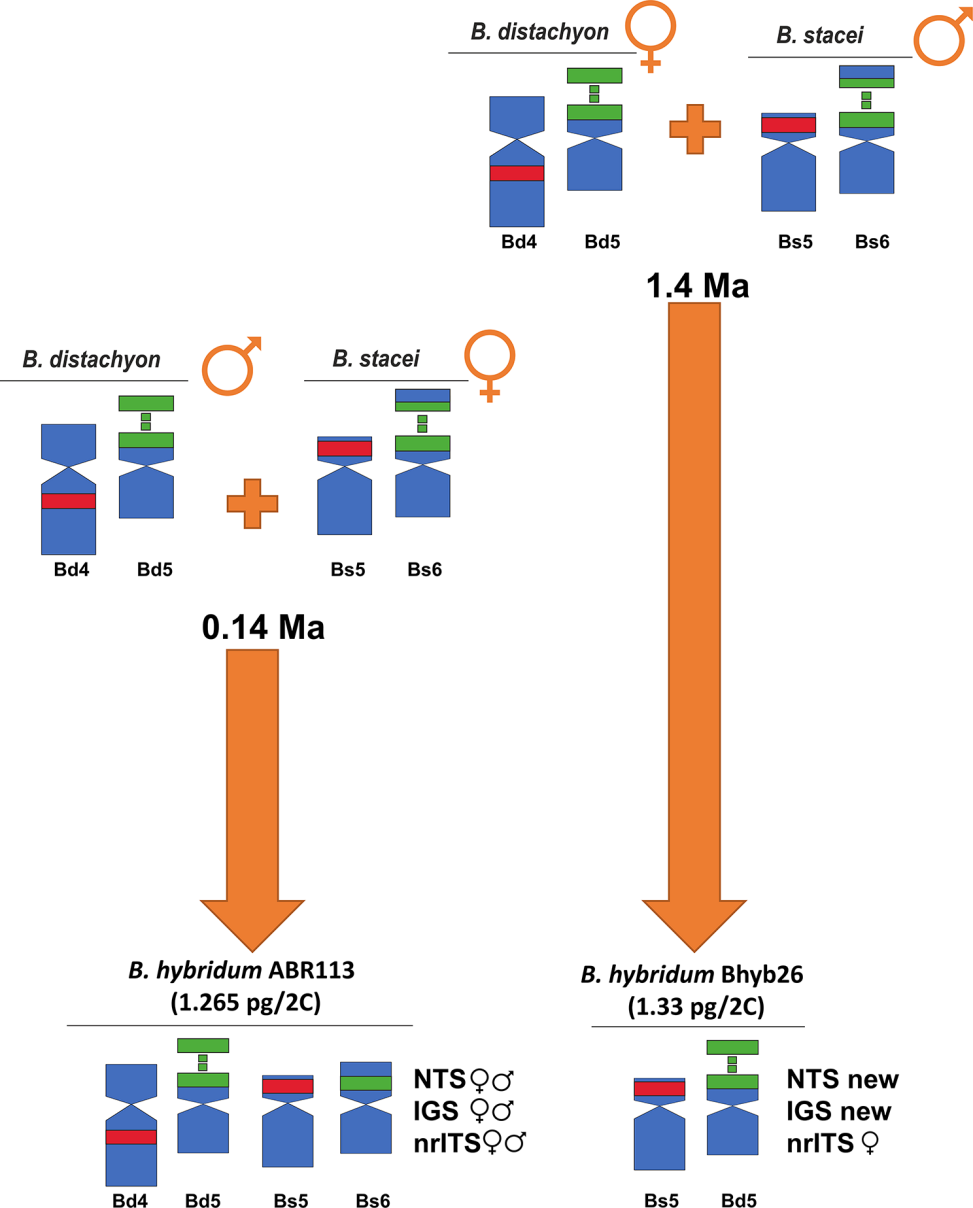
Evolution of rDNA loci in two allotetraploid lineages of *B. hybridum,* ABR113 and Bhyb26. Chromosome carrying 35S (green) or 5S (red) rDNA loci and their putative origin (♀ for maternal and ♂ for paternal parent, respectively), and types of ITS, IGS and 5S rDNA non-transcribed spacer (NTS) sequences (♀ for maternal, ♂ for paternal variants and new for a new Bhyb26-specific variant, respectively)

The presence of two distinct evolutionary lineages of *B. hybridum* representing different evolutionary ages constitutes a convenient model system for studying the fate of tandemly repetitive rRNA genes in allopolyploids. We demonstrated retention of both ancestral 35S rDNA homoeologues in the evolutionary younger ABR113 genotype, while the evolutionary older Bhyb26 genotype showed the complete elimination of the S-subgenome 35S rDNA units (Fig. 4). It is necessary to mention that the trend of gradual elimination of *B-stacei*-like rDNA units was already seen in some younger *B. hybridum* lineages [39] while the extent has never been so pronounced as in the case of Bhyb26. Uniparental loss of 35S rDNA repeats is considered a part of the diploidisation process that accompanied the evolution of plant allopolyploids. This phenomenon was also observed in different genera, e.g., *Gossypium* [59], *Triticum* [60], *Primula* [61], *Nicotiana* [62], *Atropa* [63] and *Brassica* [64]. Uniparental elimination of 35S rDNA units is not always connected with the reduction of loci number since the rDNA units of one parental subgenome can be overwritten by the units originating from the other [59]. However, uniparental loss of the entire 35S rDNA locus (or several loci) has been reported in a number of allopolyploids, e.g., *Chenopodium*, *Paspalum* and *Melampodium* [7, 65, 66] likely reflecting diploidisation processes. Well-documented examples of uniparental elimination of either maternal [7, 62] or paternal rDNA loci have been described [67, 68]. A complete elimination of 35S rDNA units from the S-subgenome (paternal parent) was observed in *B. hybridum* Bhyb26. Partial elimination of 35S rDNA units from the S-subgenome was also reported in *B. hybridum* lineages that arose from the opposite crossing direction [39]. In those cases, the 35S rDNA units were partially eliminated from the maternal subgenome. Thus, regardless of the cross direction, the 35S rDNA units were lost from the S-subgenome, indicating that the direction of the cross plays little or no role in the tendency towards rDNA elimination in *B. hybridum*.

Despite the 35S rDNA coding sequences being highly conserved, the ITS and the IGS region evolve faster, resulting in their utility in evolutionary studies [69, 70]. These regions can be species-specific, allowing the distinction of the rDNA units that belong to different subgenomes in allopolyploids since the 35S rDNA IGS interlocus homogenisation is significantly less frequent than intralocus one [71]. This is also reflected in the evolutionary patterns of rDNA in the evolutionary younger *B. hybridum* ABR113 where both the D- and S-subgenome 35S rDNA homoeologues are maintained, and no trace of interlocus homogenisation was reported [35, 36]. In contrast, in the evolutionary older *B. hybridum* Bhyb26 a different scenario has been found, with only one ancestral ITS variant (belonging to the D-subgenome one) and an emerging new IGS family (Figs. 3 and 4). This new IGS variant showed high similarity to the *B. distachyon*-like IGS. There were two motives similar to *B. stacei* and three subregions unique to Bhyb26 only (Figure S5). Our results suggested that the new IGS variant arose by reorganising the *B. distachyon*-like unit coupled with (the first, to our knowledge) rDNA intergenomic (D vs. S) recombination in the hybrid nucleus. Thus, about 1.4 Ma were needed to reconstruct the IGS by intergenomic homogenisation in Bhyb26. These observations are congruent with the hypothesis of rare homologous recombination events perhaps occurring in the nucleolus [72, 73]. Interestingly, *B. stacei* plastome-specific insertions and SNPs were also observed in the Bhyb26 D-plastotype (*B. distachyon*-type plastome) [22]. These results suggest potential S and D genomic recombinations at both nuclear and plastome levels in the ancestral Bhyb26, a finding consistent with the detection of putative heteroplasmy and chloroplast capture events in several *B. distachyon* lineages [22, 74].

5S rDNA NTS is considered a convenient marker of polyploidy in plants mainly due to its negligible level of intergenomic homogenisation [75, 76]. Several reports showed that concerted evolution operates mainly within separate 5S rDNA arrays, with little, if any, exchange between different loci [77, 78]. Usually, the 5S rDNA loci number is additive and therefore, all ancestral variants were present in allopolyploids [7, 66, 79, 80]. A similar additive pattern of 5S rDNA loci was also revealed in ABR113, where both ancestral variants of 5S rDNA NTS are present (Figs. 2 and 4). However, different evolutionary patterns of ancestral 5S rDNA homoeologues might also exist, where a significant reduction of *B. stacei-*like 5S rDNA NTS was observed in another S-plastotype *B. hybridum* accession (e.g., Bhyb127), as reflected in RepeatExplorer cluster analysis [40]. Thus, the ancestral 5S rDNAs followed different evolutionary scenarios even though both genotypes share the same cross direction and are of comparable evolutionary age [22]. Therefore, it is even more interesting when considering evolutionary older Bhyb26 (*B. distachyon* as maternal parent) where progenitor 5S rDNA types were apparently replaced by a new 5S rDNA variant (Figs. 2E-H and 4).

It is well-known that the intensity of the hybridisation signal on the chromosome is, to some extent, linked with the rDNA unit number [81]. Taking into account different FISH experiments with 5S rDNA as a probe on various *B. hybridum* genotypes, it can be assumed that at least three distinct 5S rDNA hybridisation signal patterns were observed: (i) the signals of comparable intensity in both ancestral rDNA-bearing D and S chromosomes (e.g., *B. hybridum* ABR107) [35]; (ii) the FISH signals corresponding to 5S rDNA of higher intensity in the S-subgenome locus than the D-subgenome one (e.g., *B. hybridum* ABR113 and *B. hybridum* 3-4-2; new name: Bhyb3_4_2) [35, 41]; and (iii) the 5S rDNA hybridisation signals of higher intensity in the D-subgenome than the S-subgenome one (e.g., *B. hybridum* 19-13-2; new name: Bhyb19_13_2) [35]. The differences in copy number in locus may suggest a gradual elimination of one parental variant from *B. hybridum*, which could finally lead to the elimination of the one whole ancestral 5S rDNA locus. Two different pathways of 5S rDNA diploidisation have been described up to date; one scenario involves the elimination of one entire parental locus (e.g., *Melampodium strigosum*) [66] while the second scenario implies that one ancestral variant may be overwritten by another (*Anemone baldensis*) [67, 82]. However, such an interlocus concerted evolution of 5S rDNA loci in allopolyploids seems rare since it was described only in *Anemone baldensis*. In this allohexaploid, the D-subgenome variant was replaced by the B-subgenome variant, although the 5S rDNA loci number did not change [67, 82].

Much effort has been put into deciphering the global evolutionary patterns of the ancestral subgenomes in the polyploid *B. hybridum* as it serves as a convenient model for understanding the evolutionary fates of allopolyploids. Molecular cytogenetic data [28] and the analyses of the whole genome sequences from multiple genotypes [22, 26, 27] have pointed out a lack of significant genomic rearrangements accompanying hybridisation and WGD in this species. In *Brachypodium* genomes no significant, large genomic changes have occurred during its evolution; on the contrary, there has probably been a slow, gradual process of genomic change across evolutionary time. However, rDNA sequences represent more dynamic genome components than single-copy sequences and transposable elements [22, 26, 27, 35]. Such massive changes in rDNA sequences were also described in *Arabidopsis suecica* [83], in which, as in *B. hybridum*, relative stasis in other genome components was shown. Therefore, an interesting question arises whether tandem repetitive sequences other than rDNA display such dynamic evolutionary pathways.

In conclusion, rRNA genes in *B. hybridum* demonstrate a pronounced tendency towards diploidisation, with distinct patterns of rDNA elimination observed between younger and older lineages. Notably, the 35S and 5S rDNA loci exhibit differential behaviour across subgenomes, with preferential loss of 35S rDNA from the S-subgenome and 5S rDNA from the D-subgenome in the older lineage. These results suggest that 5S and 35S rRNA genes are likely expressed from separate subgenomes in Bhyb26, highlighting the complex regulatory dynamics governing rRNA gene expression in polyploid genomes.

## Electronic supplementary material

Below is the link to the electronic supplementary material.

Supplementary Material 1

Supplementary Material 2

Supplementary Material 3

Supplementary Material 4

Supplementary Material 5

Supplementary Material 6

Supplementary Material 7

Supplementary Material 8

## Data Availability

Sequence data that support the findings of this study have been deposited in the NCBI with the primary accession code: PP339786, PP339788, PP339790, PP339792, PP339787, PP339789, PP339791, PP339793, PP339812 - PP339815, PP339808 - PP339811, PP339782 - PP339785, PP339794 - PP339807, PP317522, PP339781, PP339816 – PP339825.
