# Supplementary material for: Does time matter? Intraspecific diversity of ribosomal RNA genes in lineages of the allopolyploid model grass Brachypodium hybridum with different evolutionary ages
Source: BMC Plant Biol. 2024 Oct 18;24:981. doi: 10.1186/s12870-024-05658-5 (PMC11488067; doi:10.1186/s12870-024-05658-5)
Supplement: Supplementary file 1 — Supplementary Material 1 [file 12870_2024_5658_MOESM1_ESM.docx]

Supplementary Table S2

The GenBank accession numbers of the rDNA sequences obtained from the studied *Brachypodium* samples

| Species | 2n | x | GenBank accessions | | |
| --- | --- | --- | --- | --- | --- |
|  |  |  | **nrITS** | **IGS** | **5S NTS** |
| *B. distachyon*  Bd21 | 10 | 5 | - | - | PP339786, PP339788, PP339790, PP339792 |
| ABR5 |  |  |  |  | PP339787, PP339789, PP339791, PP339793 |
| *B. stacei*  ABR114 | 20 | 10 | - | - | PP339812 - PP339815 |
| Bsta5 |  |  |  |  | PP339808 - PP339811 |
| *B. hybridum*  ABR113 | 30 | 10+5 | - | - | PP339782 - PP339785,  PP339794 - PP339807 |
| Bhyb26 |  |  | PP317522 | PP339781 | PP339816 – PP339825 |
