# Supplementary material for: Does time matter? Intraspecific diversity of ribosomal RNA genes in lineages of the allopolyploid model grass Brachypodium hybridum with different evolutionary ages
Source: BMC Plant Biol. 2024 Oct 18;24:981. doi: 10.1186/s12870-024-05658-5 (PMC11488067; doi:10.1186/s12870-024-05658-5)
Supplement: Supplementary file 2 — Supplementary Material 2 [file 12870_2024_5658_MOESM2_ESM.docx]

Supplementary Table S1

Primers used for PCR amplification and sequencing of rDNA genes (Changet al. 2010; Maughan et al. 2006; Venora et al. 2000) in the studied *Brachypodium* samples.

| Region | Primer name and sequence (5’-3’) |
| --- | --- |
| 5S rDNA NTS | **5S forward**: GAG TAG TAC TAG GAT GGG TGA CC  **5S reverse**: ACT GCG GAG TTC TGA TGG GAT C |
| ITS1-5.8S-ITS2 | **18S dir**: CGT AAC AAG GTT TCC GTA GG  **25S com**: AGC GGG TAG TCC CGC CTG A |
| ITS1 | **18S for**: GCGCTACACTGATGTATTCAA CGA G  **5.8S rev**: CGCAACTTGCGTTCAAAGACTCGA |
| 35S rDNA IGS | **IGS_Pr1F**: TTGCTGCCACGATCCACTGAG  **IGS_Pr1R**: CTACTGGCAGGATCAACCAGG  **IGS_Pr2F**: GTCCACTCGTGACTTGTGACT  **IGS_Pr2R**: CGGACGAGGCAGGATTTCTG |
