# Supplementary figures and images for: Does time matter? Intraspecific diversity of ribosomal RNA genes in lineages of the allopolyploid model grass Brachypodium hybridum with different evolutionary ages
Source: BMC Plant Biol. 2024 Oct 18;24:981. doi: 10.1186/s12870-024-05658-5 (PMC11488067; doi:10.1186/s12870-024-05658-5)

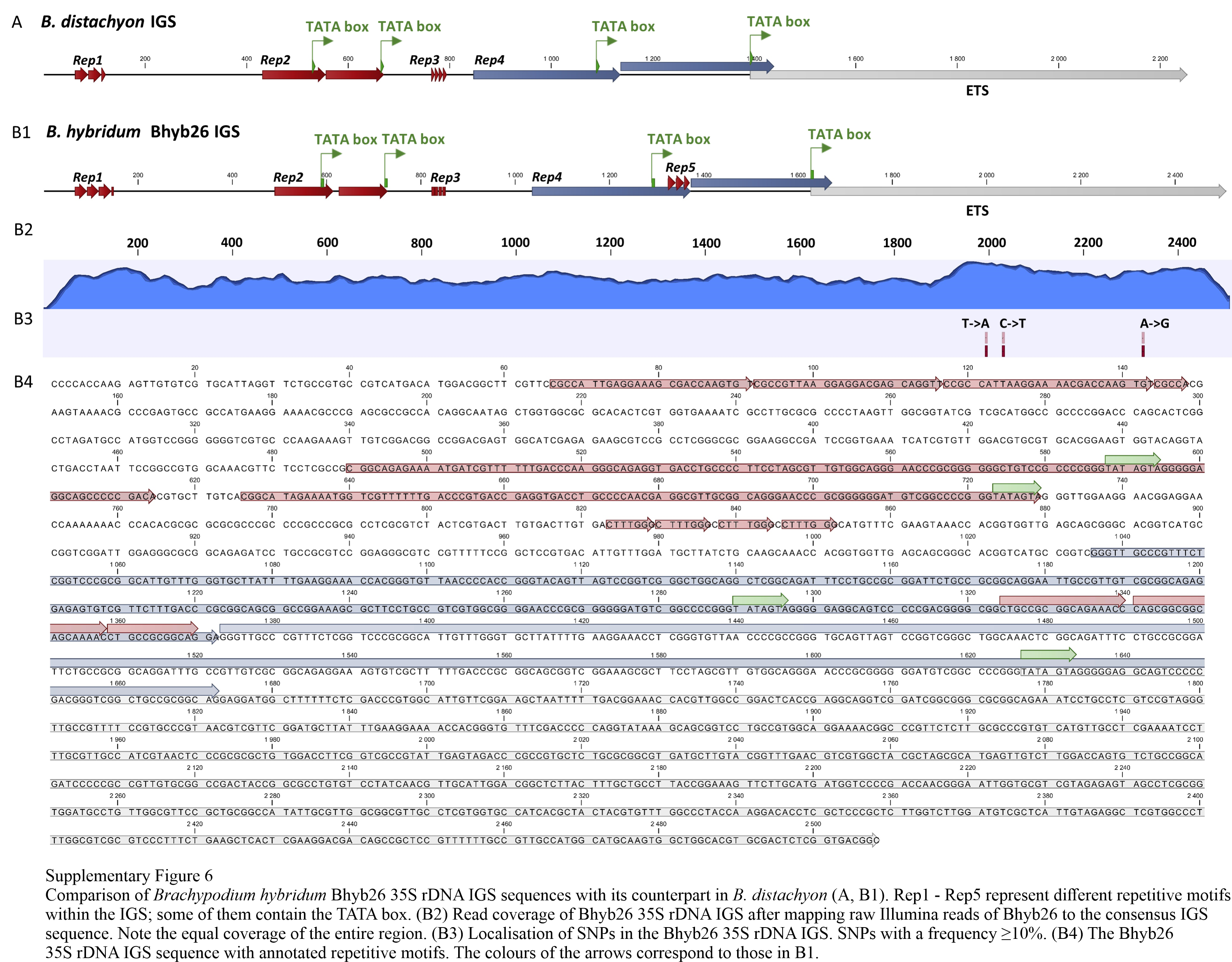

Supplement: Supplementary file 3 — Supplementary Material 3 [file 12870_2024_5658_MOESM3_ESM.tif]

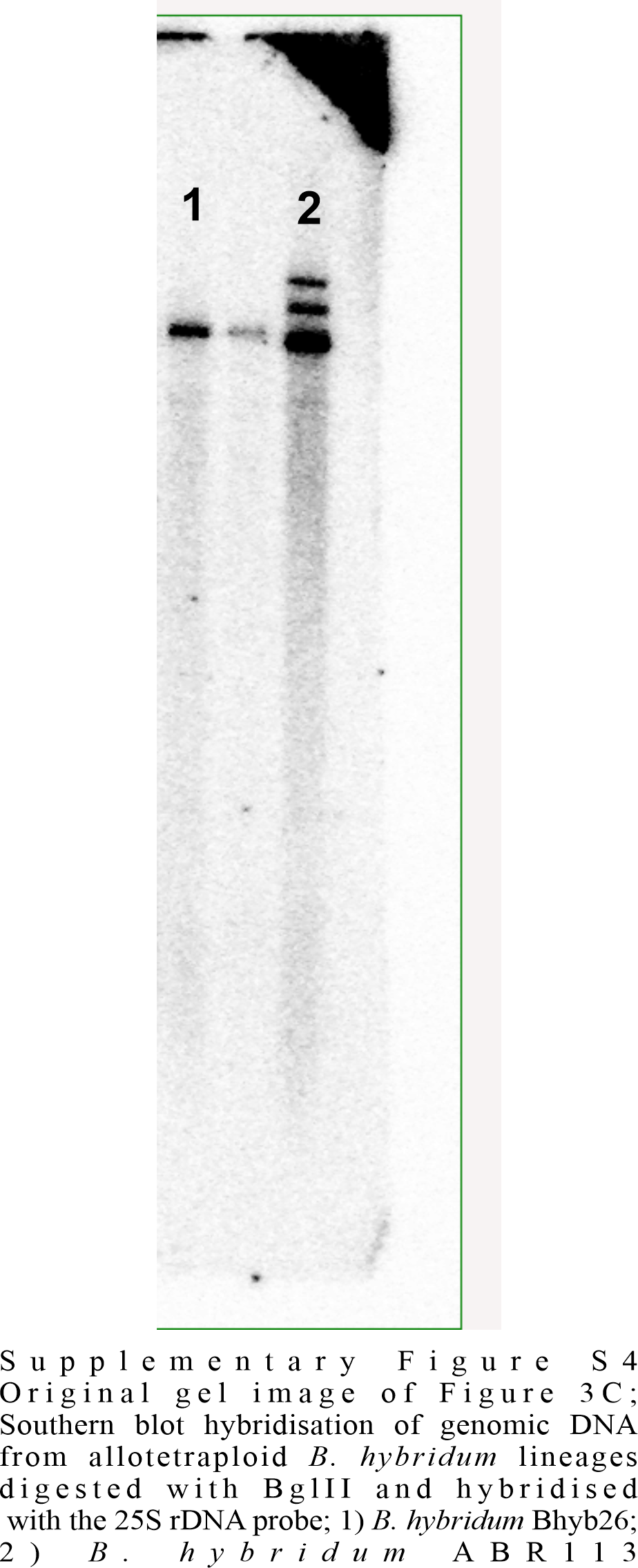

Supplement: Supplementary file 5 — Supplementary Material 5 [file 12870_2024_5658_MOESM5_ESM.tif]

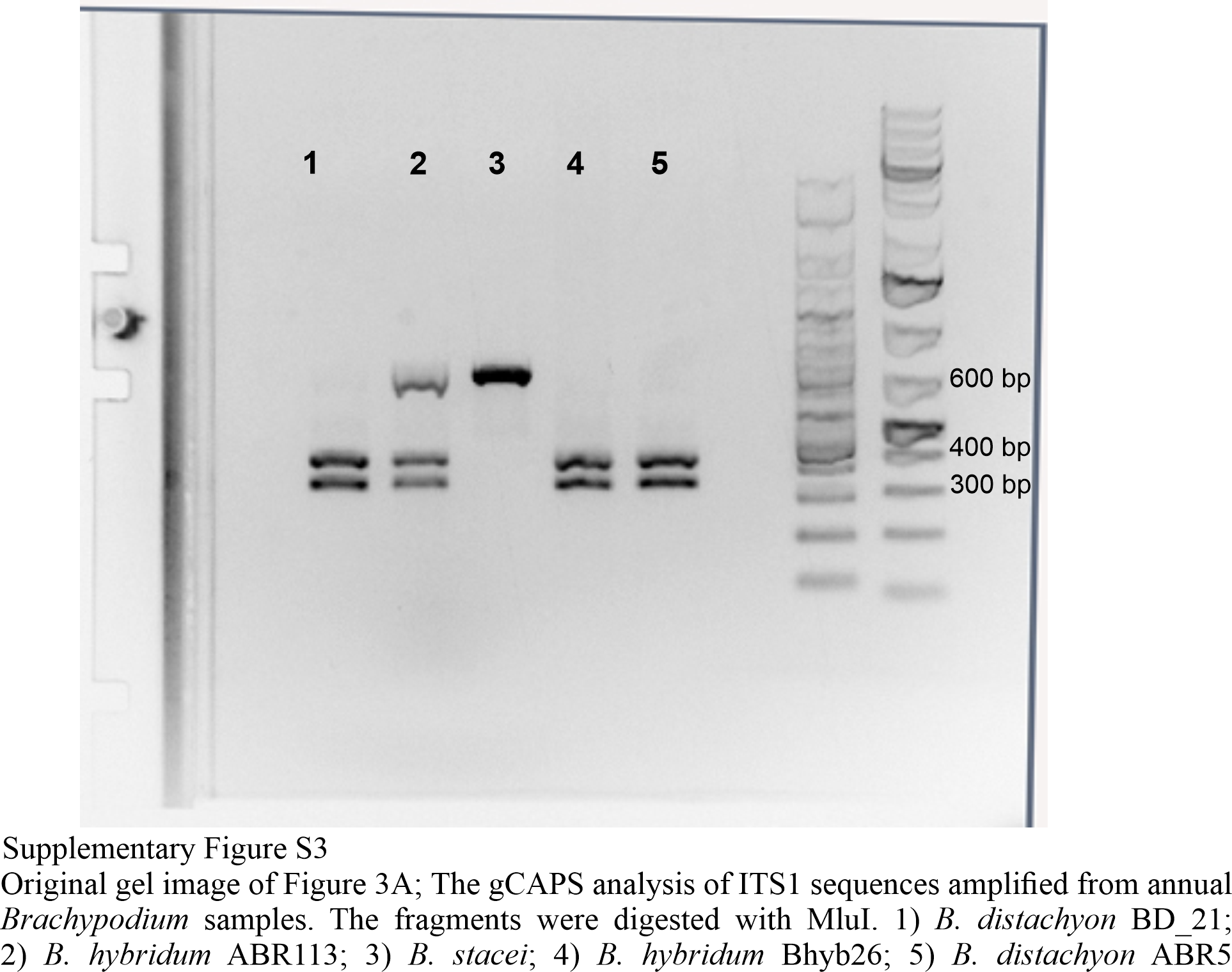

Supplement: Supplementary file 6 — Supplementary Material 6 [file 12870_2024_5658_MOESM6_ESM.tif]

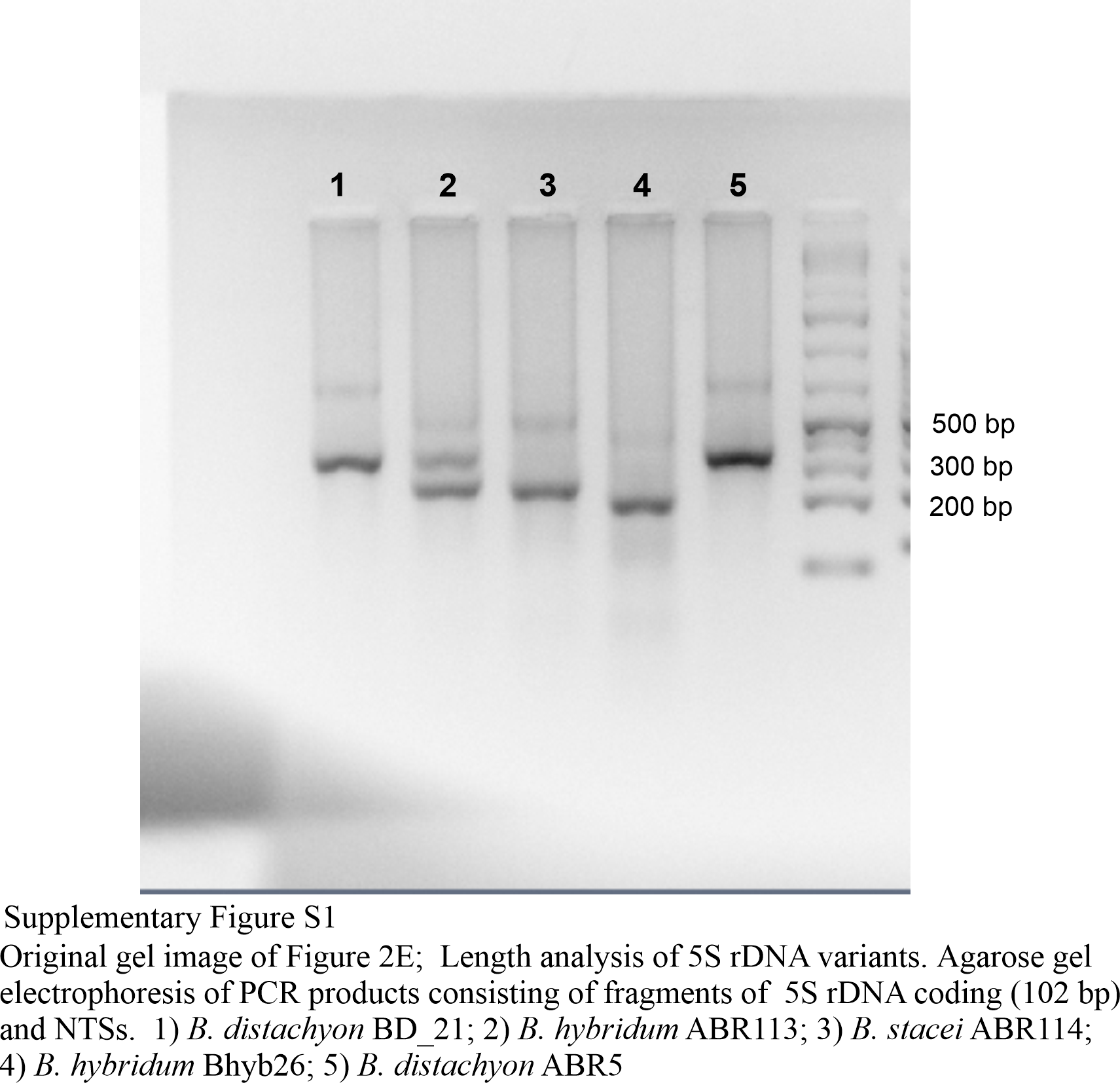

Supplement: Supplementary file 7 — Supplementary Material 7 [file 12870_2024_5658_MOESM7_ESM.tif]

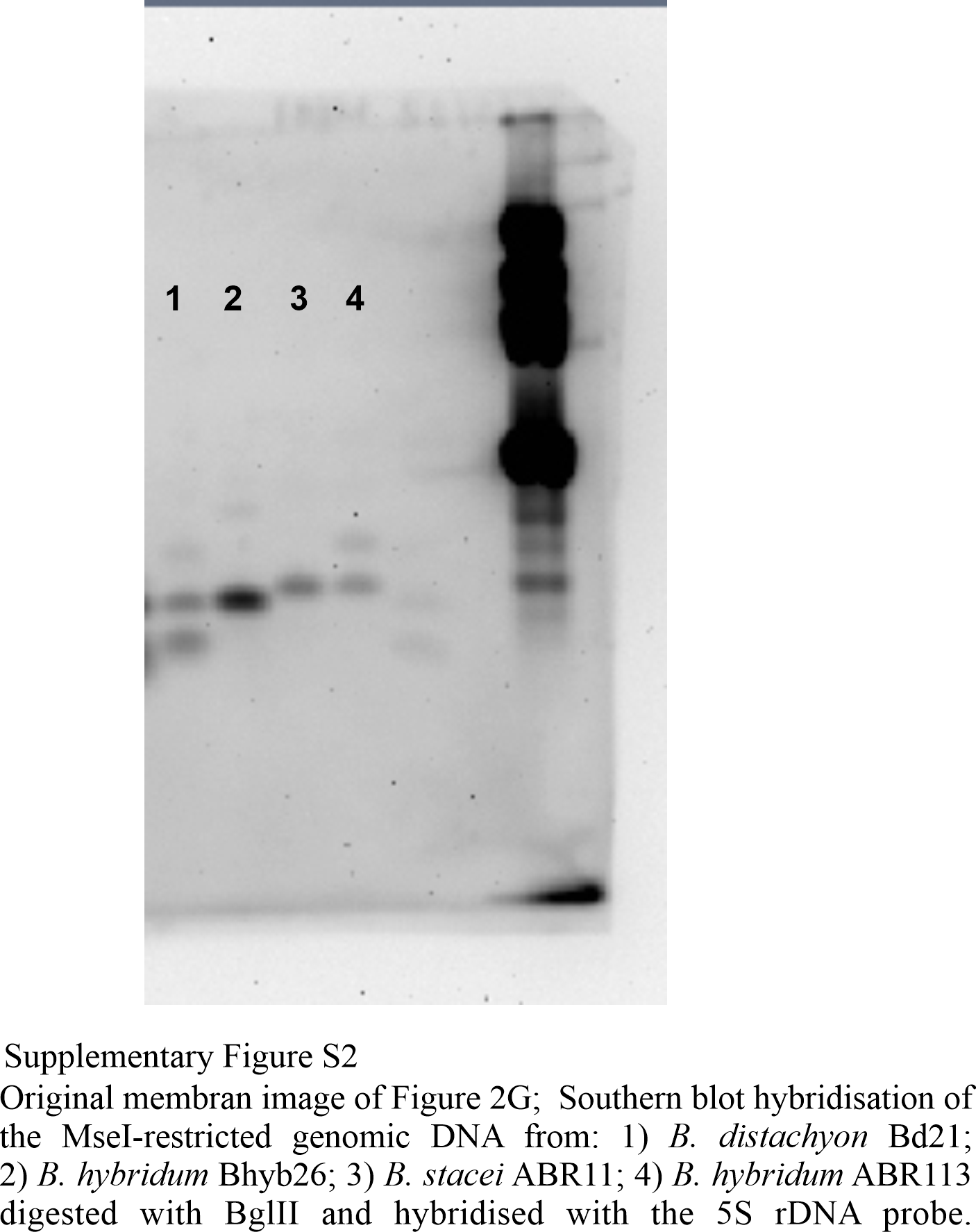

Supplement: Supplementary file 8 — Supplementary Material 8 [file 12870_2024_5658_MOESM8_ESM.tif]
