# Supplementary material for: Does time matter? Intraspecific diversity of ribosomal RNA genes in lineages of the allopolyploid model grass Brachypodium hybridum with different evolutionary ages
Source: BMC Plant Biol. 2024 Oct 18;24:981. doi: 10.1186/s12870-024-05658-5 (PMC11488067; doi:10.1186/s12870-024-05658-5)
Supplement: Supplementary file 4 — Supplementary Material 4 [file 12870_2024_5658_MOESM4_ESM.pdf]

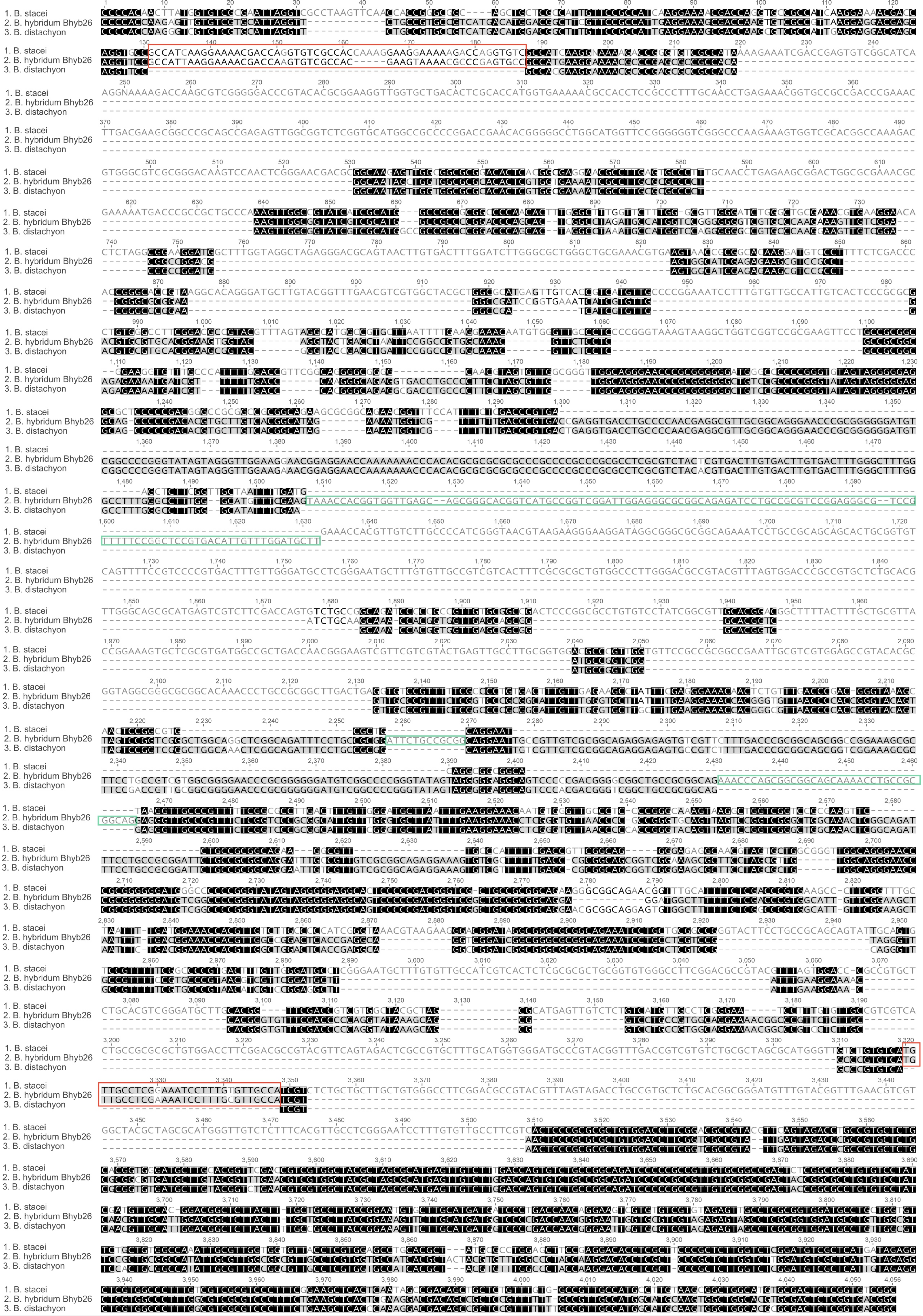

Supplementary Figure 5

Alignment of the 35S rDNA IGS of allotetraploid *Brachypodium hybridum* Bhyb26 and diploids *B. distachyon* and *B. stacei*. Grey background and red and green frames indicate regions similar to *B. distachyon* and *B. stacei* and unique to Bhyb26, respectively.
